# T Cells Immunology in the Immunological Diseases

**DOI:** 10.1155/2014/690324

**Published:** 2014-09-01

**Authors:** Xiuli Wu, Grzegorz K. Przybylski, Qintai Yang, Qifa Liu

**Affiliations:** ^1^Institute of Hematology, Medical College, Jinan University, Guangzhou 510632, China; ^2^Institute of Human Genetics, Polish Academy of Sciences, 60-479 Poznań, Poland; ^3^Department of Otolaryngology, Yong Loo Lin School of Medicine, National University of Singapore, Singapore 119228; ^4^Department of Hematology, Nanfang Hospital, Southern Medical University, Guangzhou 510515, China

Immunological diseases, with the morbidity keeping increased, have become a major threat to human mental and physical health. However, the pathogenesis of these diseases is extremely complicated and remains unclear. Recent advances in biology have introduced new technologies to study the underlying mechanisms that contribute to the development of immunotherapy of immunological diseases. This special issue includes original research articles and review articles that do research on T cells immunology in the immunological diseases.

In the brief review “*The pathology of T cells in systemic lupus erythematosus*” by A. Mak and N. Y. Kow, the authors discuss a detailed account of the putative mechanisms by which the normal physiology of T cells are disturbed and why do the regulatory T cells fail to alleviate proinflammatory response in systemic lupus erythematosus (SLE) and introduce the current state of clinical trials evaluating therapeutic agents which target molecules expressing on and inside T cells for the treatment of SLE.

In the review “*The role of the* *γ* *δ* *T cell in allergic diseases*” by R. Zheng and Q. Yang, *γ* *δ* T cells have been considered to bridge the innate and adaptive immunity. *γ* *δ* T cells may play crucial roles in the development and perpetuation of allergic inflammation as effector and immunoregulatory cells. This review focuses on the latest knowledge on characteristics and roles of *γ* *δ* T cells in allergic diseases.

The review “*Improving cytomegalovirus-specific T cell reconstitution after haploidentical stem cell transplantation*” by X. Luo et al. summarizes the kinetics of cytomegalovirus- (CMV-) specific T cell recovery and its association with CMV infection after haploidentical stem cell transplantation and discusses the strategies to improve CMV-specific immune reconstitution.

In the research “*Alternative expression pattern of MALT1-A20-NF-*κ*B in patients with rheumatoid arthritis*” by X. Wang et al., rheumatoid arthritis (RA) is a common, chronic, systemic, and inflammatory autoimmune disorder and abnormal T cell immunity plays a critical role in the development of RA. Recently, a negative regulator of nuclear factor kappa B (NF-*κ*B), A20, was identified as a key regulator for T cell activation and inflammatory signaling and may be involved in RA pathogenesis. This study analyzed the expression level of A20, NF-*κ*B, and the A20 regulatory factor mucosa-associated-lymphoid-tissue lymphoma-translocation gene 1 (MALT1) in patients with RA. The authors characterized the alternative expression pattern of MALT1, A20, and NF-*κ*B in RA, which might be related to abnormal T cell activation. The lack of A20 and dysfunctional MALT1 are common features in Chinese patients with RA, and the results provide new data for the consideration of target regulation in RA inflammation.

In the research “*Rapamycin regulates iTreg function through CD39 and Runx1 pathways*” by Y. Lu et al., the induced regulatory T cells (iTreg) play important roles in treating various autoimmune diseases in mice including autoimmune diabetes, experimental arthritis and other immune-mediated inflammatory diseases. CD39 is a newly determined Treg marker that relates to cell suppression. Runx1, a regulator of FoxP3, controls the expression of adenosine deaminase gene, which is found recently in the downstream of CD39 pathway in trophoblast cells. The authors suggested that CD39 expression was involved in iTreg generation and the enhanced suppressive ability of rapamycin induced Treg was partly due to Runx1 pathway. This study provides a novel insight into the mechanisms of iTreg generation enhanced by rapamycin.

In the research “*The feature of distribution and clonality of TCR* *γ*/*δ* *subfamilies T cells in patients with B-cell non-Hodgkin lymphoma*” by L. Wang et al., T-cell immunodeficiency is a common feature in cancer patients, which may contribute to the disease initiation and progression. This study provides a preliminary profile of distribution and clonality of T-cell receptor *γ*/*δ* subfamilies T cells in peripheral blood (PB), bone marrow (BM), and lymph node (LN) from B-cell non-Hodgkin lymphoma patients. The clonally expanded V*δ*5, V*δ*6, and V*δ*8 subfamily T cells were detected only in PB but neither in BM nor in LN. While clonally expanded V*δ*2 and V*δ*3 T cells could be detected in both PB and BM or PB and LN. Similar clonally expanded V*δ* subfamily T cells in PB and BM may be related to the same B-cell lymphoma-associated antigens, while the different reactive clonally expanded V*γ*/V*δ* T cells may be due to local immune response.

*Xiuli Wu* *Xiuli Wu* *Grzegorz K. Przybylski* *Grzegorz K. Przybylski* *Qintai Yang* *Qintai Yang* *Qifa Liu* *Qifa Liu*

